# Supplementary material for: Different Growth and Sporulation Responses to Temperature Gradient among Obligate Apomictic Strains of Ulva prolifera
Source: Plants (Basel). 2021 Oct 22;10(11):2256. doi: 10.3390/plants10112256 (PMC8617885; doi:10.3390/plants10112256)
Supplement: Supplementary file 1 [file plants-10-02256-s001.zip › plants-1421601-supplementary.pdf]

Article

Different Growth and Sporulation Responses to Temperature Gradient among Obligate Apomictic Strains of *Ulva prolifera*

Yoichi Sato [1,2,*], Yutaro Kinoshita [1,3], Miho Mogamiya [1], Eri Inomata [1], Masakazu Hoshino [4] and Masanori Hiraoka [3,*]

[1] Bio-Resources Business Development Division, Riken Food Co., Ltd., Miyagi 985-0844, Japan; yuu_kinoshita@rikenfood.co.jp (Y.K.); mih_mogamiya@rikenfood.co.jp (M.M.); eri_inomata@rikenfood.co.jp (E.I.)
[2] Nishina Center for Accelerator-Based Science, RIKEN, Saitama 351-0198, Japan
[3] Usa Marine Biological Institute, Kochi University, Kochi 781-1164, Japan
[4] Department of Algal Development and Evolution, Max Planck Institute for Developmental Biology, Max-Planck-Ring 5, 72076 Tübingen, Germany; mhoshino.sci@gmail.com
* Correspondence: yoi_sato@rikenfood.co.jp (Y.S.); mhiraoka@kochi-u.ac.jp (M.H.); Tel.: +81-22-395-4226 (Y.S.); +81-88-856-0426 (M.H.)

Supplementary Materials

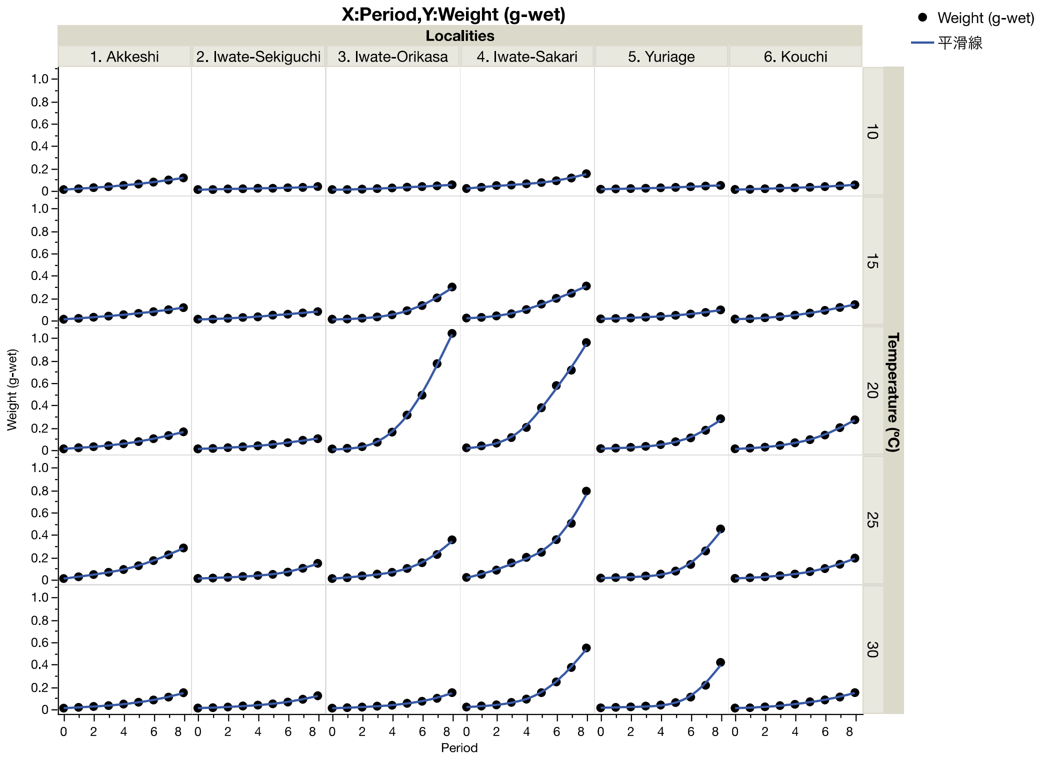

(a)

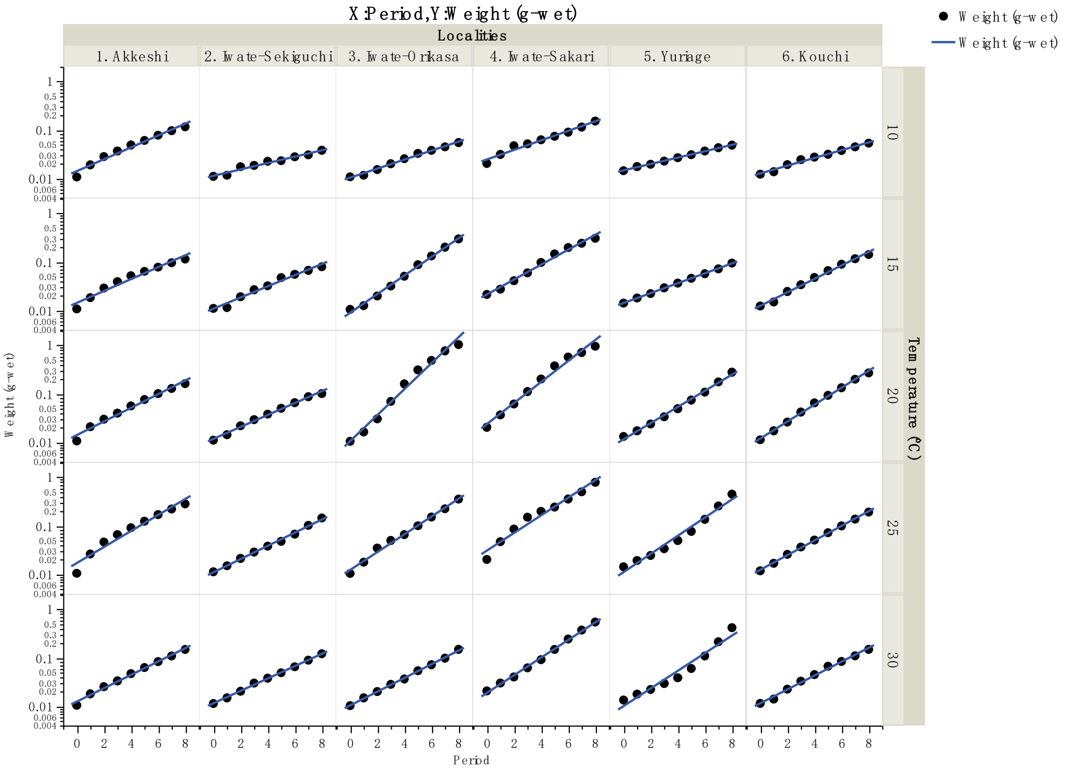

(b)

Figure S1. Upper figure: Wet weight variations in thallus clusters of six strains of *Ulva prolifera* incubated in vitro at one of five different temperatures, 150 µmol photons m^{-2} s^{-1} (12/12 h light/dark cycle) in half-strength ES medium with aeration for 8 d. Lower figure: the same data expressed as natural logarithms. The slopes for each combination of strain and temperature were used to generate the relative growth rate values in Figure 2.

Table S1. Differences in RGR, Carbon, and nitrogen contents among five strains of Ulva prolifera at 10, 15, 20, 25, and 30 °C. Data were analyzed using Kruskal–Wallis test followed by post-hoc Scheffe's test for multiple comparisons.

Temperature	Contents	Kruskal-Wallis test			Strain	Multiple Comparison test				
		$\chi2$	*df*	p		1	2	3	4	5
					1	–				
					2	n.s.	–			
	RGR	13.97	5	0.016	3	n.s.	n.s.	–		
					4	n.s.	n.s.	n.s.	–	
					5	**	n.s.	n.s.	n.s.	–
					1	–				
10					2	n.s.	–			
	Carbon	14.85	5	0.011	3	n.s.	n.s.	–		
					4	n.s.	n.s.	n.s.	–	
					5	n.s.	n.s.	n.s.	n.s.	–
					1	–				
	Nitrogen	13.25	5	0.021	2	n.s.	–			
					3	n.s.	n.s.	–		
					4	n.s.	n.s.	n.s.	–	
					5	n.s.	n.s.	n.s.	n.s.	–
					1	–				
					2	n.s.	–			
	RGR	11.05	5	0.051	3	n.s.	n.s.	–		
					4	n.s.	n.s.	n.s.	–	
					5	n.s.	n.s.	n.s.	n.s.	–
					1	–				
15					2	n.s.	–			
	Carbon	13.30	5	0.021	3	n.s.	n.s.	–		
					4	n.s.	n.s.	n.s.	–	
					5	n.s.	n.s.	n.s.	n.s.	–
					1	–				
	Nitrogen	10.24	5	0.069	2		–			
					3			–		
					4				–	
					5					–
					1	–				
					2	n.s.	–			
	RGR	20.05	5	0.001	3	n.s.	*	–		
					4	n.s.	n.s.	n.s.	–	
					5	n.s.	n.s.	n.s.	n.s.	–
					1	–				
20					2	n.s.	–			
	Carbon	13.02	5	0.023	3	n.s.	n.s.	–		
					4	n.s.	n.s.	n.s.	–	
					5	n.s.	n.s.	n.s.	n.s.	–
					1	–				
	Nitrogen	14.43	5	0.013	2	n.s.	–			
					3	n.s.	n.s.	–		
					4	n.s.	n.s.	n.s.	–	
					5	n.s.	n.s.	n.s.	n.s.	–
					1	–				
					2	n.s.	–			
	RGR	6.84	5	0.233	3	n.s.	n.s.	–		
					4	n.s.	n.s.	n.s.	–	
					5	n.s.	n.s.	n.s.	n.s.	–
					1	–				
25					2	n.s.	–			
	Carbon	14.75	5	0.012	3	n.s.	n.s.	–		
					4	n.s.	n.s.	n.s.	–	
					5	n.s.	n.s.	n.s.	n.s.	–
					1	–				
	Nitrogen	16.25	5	0.006	2	n.s.	–			
					3	n.s.	n.s.	–		
					4	n.s.	n.s.	n.s.	–	
					5	n.s.	n.s.	n.s.	n.s.	–
30	RGR	8.04	5	0.154	1	–				

				2	n.s.	–			
				3	n.s.	n.s.	–		
				4	n.s.	n.s.	n.s.	–	
				5	n.s.	n.s.	n.s.	n.s.	–
				1	–				
				2	n.s.	–			
Carbon	14.59	5	0.012	3	n.s.	n.s.	–		
				4	n.s.	n.s.	n.s.	–	
				5	n.s.	n.s.	n.s.	n.s.	–
				1	–				
				2	n.s.	–			
Nitrogen	15.69	5	0.008	3	n.s.	n.s.	–		
				4	n.s.	n.s.	n.s.	–	
				5	n.s.	n.s.	n.s.	n.s.	–

* $p<0.05$; ** $p<0.01$; n.s.: no significance.
